# The association between high‐risk human papillomavirus and oral lichen planus

**DOI:** 10.1002/cre2.707

**Published:** 2023-01-13

**Authors:** Maryam Mohammadi, Hamid Abbaszadeh, Nooshin Mohtasham, Hamid Salehiniya, Ebrahim Shafaie

**Affiliations:** ^1^ Student Research Committee Birjand University of Medical Sciences Birjand Iran; ^2^ Department of Oral and Maxillofacial Pathology, Faculty of Dentistry Birjand University of Medical Sciences Birjand Iran; ^3^ Oral and Maxillofacial Disease Research Center, Faculty of Dentistry Mashhad University of Medical Science Mashhad Iran; ^4^ Social Determinants of Health Research Center Birjand University of Medical Sciences Birjand Iran; ^5^ Infectious Diseases Research Center Birjand University of Medical Sciences Birjand Iran

**Keywords:** human papillomavirus 16, human papillomavirus 18, oral lichen planus

## Abstract

**Objectives:**

Oral lichen planus (OLP) is a cell‐mediated inflammatory mucosal disorder and is classified as an oral potentially malignant disorder. Some research has shown that apoptosis in OLP cells is similar to a viral infection such as human papillomavirus (HPV). So, the aim of this case‐control study was to investigate the association of high‐risk HPV with OLP.

**Material and Methods:**

DNA was extracted from 25 formalin‐fixed, paraffin‐embedded (FFPE) OLP tissues and 25 FFPE normal oral tissues as case and control groups, respectively. The presence of high‐risk HPV16 and HPV18 DNA was investigated by polymerase chain reaction (PCR). *p*‐value<.05 was considered significant.

**Results:**

Twelve samples (48%) of OLPs were positive for HPV16, compared with six samples (24%) of controls; although the difference was not significant, it was borderline (*p* = .07). Three samples (12%) of OLPs were positive for HPV18 compared with one sample (4%) of controls; the difference was not significant (*p* = .3). The total frequency of both high‐risk HPV were 14 samples (56%) of OLPs and 7 samples (28%) of controls; there was a significant association between the high‐risk HPV and OLP (*p* = .04). High‐risk HPVs was more prevalent in erosive‐atrophic (EA) form of OLP as compared to non‐EA form, although the difference was not significant (*p* = .13).

**Conclusions:**

The results suggest a significant association between high‐risk HPVs and OLP.

## 1 INTRODUCTION

Oral lichen planus (OLP) is a chronic inflammatory mucocutaneous disorder, which reveals atrophy or thickening of the epithelial with or without ulcers in 0.5%–2% of the general population, depending on geographical differences (Boorghani et al., 2010; Gupta & Jawanda, 2015; Ma et al., 2016). OLP is diagnosed based on clinical and histopathological criteria (Warnakulasuriya et al., 2021). Even though the World Health Organization classifies OLP as an oral potentially malignant disorder (OPMD), the association between OLP and the potential precancerous condition remains controversial since less than 1% of cases of OLP progress to malignancy (Georgakopoulou et al., 2012; Y. Liu et al., 2010). All types of OLP can be divided into two clinical groups: erosive atrophic form (EA‐OLP) and non‐erosive atrophic form (non‐EA‐OLP; Ma et al., 2016). OLP is more commonly reported in the middle‐aged and is more common in women (Boorghani et al., 2010). It is generally accepted that OLP is a T‐cell‐mediated inflammatory disease, but the exact cause of OLP is still unknown; various studies have shown risk factors including genetics (Ognjenović et al., 1998), drugs (Fortuna et al., 2017), infectious agents (Alves et al., 2011; Gorsky & Epstein, 2011; Yildirim et al., 2011), autoimmunity (Abbate et al., 2006), trauma (Scully et al., 1998), diabetes, and hypertension (Ahmed et al., 2012). Some research has shown that apoptosis in OLP cells is similar to a viral infection, in which the virus acts as a cytoplasmic antigen and causes the host cell protein profile deformation (Shukla, 2014). Recent investigations support the association of the human papillomavirus (HPV) with OLP etiopathogenesis (Ma et al., 2016). The oral HPV prevalence is 6.1% in Iranian healthy people, among these, high‐risk strains, HPV‐18 and HPV‐16 are the most common (Seifi et al., 2013). The proven role of HPV in cervical cancer and oropharyngeal squamous cell carcinoma (SCC) has been hypothesized that HPV, especially its high‐risk strains, may also play a role in the development of OLP (Neville et al., 2016). Due to disagreement on this issue, the aim of this case‐control study was to investigate the association of high‐risk HPV with OLP.

## 2 MATERIALS AND METHODS

This study was approved by the ethics committee of the University (ethics code: IR.BUMS.REC.1399.316).

### 2.1 Tissue collection

Twenty‐five formalin‐fixed paraffin‐embedded (FFPE) tissues of confirmed OLP (11 EA‐OLPs and 14 non‐EA‐OLPs) were obtained from the oral pathology department as the case group. Another 25 FFPE tissue specimens were obtained as a control group from patients referred for wisdom tooth surgery; the case and control groups were age and sex‐matched. The OLP specimens were examined by two experienced oral pathologists.

### 2.2 DNA extraction

The DNA of all 50 paraffin‐embedded samples was extracted with a high pure polymerase chain reaction (PCR) template preparation kit (Cat. no: 11796828001, Roche Diagnostics GmbH, Penzberg, Germany), according to the manufacturer's instructions for FFPE tissue specimens. The quality and quantity of extracted DNA were evaluated by NanoDrop 2000 (Thermo Fisher Scientific, Waltham, United States of America) and stored at −20°C for molecular applications.

### 2.3 PCR assay

PCR was used to investigate the presence of HPV16 and HPV18 in 200 ng of DNA template by previously described primers (Table 1).

**Table 1:** Sequences of type‐specific HPV primers

Primer	Primer sequence	Amplicon size (bp)	References
HPV‐18	F: 5′‐CACTTCACTGCAAGACATAGA‐3′ R: 5′‐GTTGTGAAATCGTCGTTTTTCA‐3′	322	Khabaz (2016)
HPV‐16	F: 5′‐TCAAAAGCCACTGTGTCCTGA‐3′ R: 5′‐CGTGTTCTTGATGATCTGCAA‐3′	120	Syrjänen et al. (1991)

**Figure 1:**
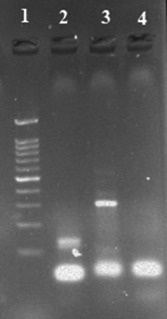
HPV type‐specific polymerase chain reaction (PCR); 1: DNA marker (1 kb), 2: HPV‐16 PCR product (120 bp), 3: HPV‐18 PCR product (322 bp), and 4: Negative control. HPV, human papillomavirus.

Each PCR reaction contained 12.5 µl of 2× Taq DNA polymerase Master Mix Red (Amplicon, Denmark), 1 µl of each 10 pM/µl forward and reverse primers (Metabion GmbH, Germany), 200 ng of DNA template, and Double Distilled water up to 25 µl final volume. 2× universal gradient thermocycler (PEQLAB, GmbH, Germany) was used for DNA amplification, starting with the initial denaturation step for 5 min at 94°C, followed by 35 cycles of denaturation at 94°C for 30 s, annealing at 56°C for 30 s and extension at 72°C for 45 s. In the end, the final extension was completed at 72°C for 10 min. HPV16 and HPV18 genomic DNA were used as positive controls. An image was taken of the PCR products under UV light following 2% agarose gel electrophoresis. The PCR product size (bp) was determined by comparing it with 1 kb DNA ladder (Figure 1).

### 2.4 Statistical analysis

All statistical analyses were run using the SPSS software version 22 (Chicago, United States of America). Associations were determined by using the Chi‐Square test or Fisher's exact test. Statistical significance was assumed if *p* < .05. All reported *p* values are two‐sided.

## 3 RESULTS

Table 2 summarizes the demographic data of the studied samples (Table 2). 56% of OLP specimens were of the non‐erosive‐atrophic (non‐EA) subtype, and 44% were of erosive‐atrophic (EA) subtype.

**Table 2:** Study characteristics

Group variable	Control, *n* (%)	Oral lichen planus, *n* (%)		*p* value
**Gender**				
Male	13 (52%)	12 (48%)		.77
Female	12 (48%)	13 (52%)		
**Age (mean ± SD)**	44.08 ± 4.28	48.28 ± 6.33		.11
**Type of lesion**	Normal oral tissue	Non‐erosive‐atrophic	Erosive‐atrophic	
	25 (100%)	14 (56%)	11 (44%)	–

Twelve of 25 (48%) of OLP tissue samples were positive for HPV16, compared with 6 of 25 (24%) of normal control tissue samples; although there was not any significant difference between studied groups based on the frequency of HPV16, the difference was borderline (*p* = .07). Our results showed the frequency of HPV16 in the OLP group was twice as high as the control group (Table 3).

**Table 3:** Prevalence of high‐risk human papillomavirus (HPV) DNA in oral lichen planus and control groups

Group HPV subtype	Oral lichen planus, *N* (%)	Control, *N* (%)	*p* value
**HPV16**			
Not present	13 (52%)	19 (76%)	.07
present	12 (48%)	6 (24%)	
**HPV18**			
Not present	22 (88%)	24 (96%)	.305
Present	3 (12%)	1 (4%)	
**HPV16 and HPV18**			
Not present	11 (44%)	18 (72%)	.04
Present	14^a^ (56%)	7 (28%)	

^a^ Simultaneous presence of HPV16 and HPV18 was detected in a sample of the oral lichen planus group.

On the other hand, 3 of 25 (12%) of OLP tissue samples were positive for HPV18, compared with 1 of 25 (4%) of normal control tissue samples; there was not any significant difference between studied groups based on the frequency of HPV18 (*p* = .3). However, the total frequency of both HPV16 and HPV18 were 14 of 25 (56%) and 7 of 25 (28%) in OLP and normal control tissue, respectively; there was a significant association between the presence of high‐risk HPVs and OLP (*p* = .04). Simultaneous presence of HPV16 and HPV18 was detected in a sample of the non‐EA OLP subgroup.

There was no significant association between the prevalence of high‐risk HPVs in studied groups based on gender (Table 4).

**Table 4:** Prevalence of high‐risk human papillomavirus (HPV) DNA based on sex in oral lichen planus and control groups

Group HPV subtype	Control			Oral lichen planus		
	Men, *N* (%)	Women, *N* (%)	*p* value	Men, *N* (%)	Women, *N* (%)	*p* value
HPV16	5 (38.5)	1 (8.3)	.41	6 (50)	6 (46)	.85
HPV18	0	1 (8.33)	1	2 (16.6)	1 (7.6)	.59
HPV16 and HPV18	5 (38.5)	2 (16.6)	.22	7^a^(58.3)	7 (53.8)	.82

^a^ Simultaneous presence of HPV16 and HPV18 was detected in a sample of OLP group .

Comparison of the OLP subgroups showed that the high‐risk HPVs were more prevalent in EA‐OLP as compared to non‐EA, although no statistically significant association was found (*p* = .13; Table 5).

**Table 5:** Prevalence of high‐risk human papillomavirus (HPV) DNA in erosive‐atrophic and non‐erosive‐atrophic forms of oral lichen planus (OLP)

OLP subtype HPV subtype	Non‐erosive‐atrophic, *N*/total (%)	Erosive‐atrophic, *N*/total (%)	*p* value
HPV16	6/14 (42.8)	6/11 (54.5)	.56
HPV18	1/14 (7)	2/11 (18.2)	.40
HPV‐16 and HPV‐18	6/14^a^ (42.8)	8/11 (72.7)	.13

^a^ Simultaneous presence of HPV16 and HPV18 was detected in a sample of lichen planus group.

## 4 DISCUSSION

This study aimed to investigate the association of high‐risk HPV with OLP; although the difference in HPV16 prevalence in OLP and control groups was not significant, it was borderline. The difference in HPV18 prevalence in the studied groups was not significant. However, there was a significant association between both high‐risk HPVs and OLP.

A significant percentage of oral cancers are preceded by visible oral mucosal changes as white or red patches (namely OPMDs). A strong association between HPV infection and OPMD development has been proposed. OLP is a chronic cell‐mediated inflammatory mucosal disorder and is classified as OPMD (McCartan & Healy, 2008). So, HPV detection and genotype determination are of great clinical importance, especially for cancer prevention and diagnosis (Poulopoulos et al., 2017).

In fact, confirming a definitive role for HPV in the development or malignant transformation of OLPs will support virus screening, HPV vaccination, and antiviral therapy combined with conventional treatments in OLP patients (along with improvement in OLP prognosis; Vijayan et al., 2021).

The role of HPV as a potential factor in the OLP development and progression of malignant transformation has been examined in some studies. There is a considerable variation reported on HPV prevalence in OLP in different parts of the world. The results of our study showed that the cumulative frequency of the high‐risk HPVs was significantly higher in the OLP group as compared with the normal control group. HPV16 was a more frequent genotype (85%) in both OLP and control groups. Also, the frequency of HPV16 in the OLP group was two times as high as in the control group. In a systematic review study by Syrjanen et al. (2011), the overall prevalence of HPV was significantly higher in OLP patients than in the healthy control; this correlation was greater in the HPV16 genotype. Our result is in line with Gonzalez et al. (2007) study that a statistically significant association was observed between the presence of HPV and OLP samples by PCR method. In the present study, the HPV16 and HPV18 DNA were detected in 6/25 (24%) and 1/25 (4%) of the normal control group, respectively. The pooled HPV detection rate is 13% of normal oral mucosa based on a previous systematic review study (Syrjänen et al., 2011). However, in some studies, there was no evidence of HPV presence in the normal oral mucosa (Campisi et al., 2004; González et al., 2007; Sameera et al., 2019; Sand et al., 2000). In Sand et al.'s (2000) study, HPV DNA was detected in 27% and 0% in the OLP and healthy control groups, respectively; in the Swedish cohort, 83% of OLP samples were positive for HPV18 by PCR method. In another study on the Indian population, the HPV18 detection rates were 86% of OLP samples and 0% of controls (Sameera et al., 2019). Older studies have found no significant association between the presence of HPV and OLP (Miller et al., 1993; Young & Min, 1991). In the present study, no significant association was found in HPV prevalence in OLP and control groups based on gender. Our result is in line with Della Vella et al.'s (2021) study, and differed from the results of Sameera et al.'s (2019) study. The present study showed that the high‐risk HPVs were more prevalent in EA‐OLP as compared to non‐EA, although no statistically significant relationship was found. EA‐OLP has more malignant potential than non‐EA OLP. In a study conducted by Campisi et al. (2004), HPV DNA was found in 18.5% of non‐EA OLP and 20.4% of EA OLP, without any significant association. In a study by Mattila et al. (2012), HPV was detected in 15.9% of EA‐OLP samples, but the importance of the presence of HPV in EA‐OLP was somewhat obscure due to the predominance of low‐risk HPV genotypes.

According to the contradictory results obtained from previous studies regarding the association between HPV infection and OLP, in recent years (especially the last 5 years), more and more studies have focused on investigating this association. A summary of these studies is presented in Table 6 to get a better understanding of the current situation in this field (Table 6). Overall, the detection rate of HPV varied from 0%–86.6% in OLP samples and 0%–28% in control (normal oral mucosa) samples in these studies. There are still conflicting results about the association between HPV infection and OLP; while some report the high detection rate (DR) of HPV virus in OLP lesions or the significant difference in the frequency of the virus between OLP and normal mucosa (Della Vella et al., 2021; Farhadi et al., 2020; Kaewmaneenuan et al., 2021; T. Liu et al., 2018; Poulopoulos et al., 2017; Sameera et al., 2019; Vijayan et al., 2022), others report the low detection rate of the virus or the nonsignificant difference in the frequency of the virus between OLP and normal mucosa (Bar et al., 2021; Gomez‐Armayones et al., 2019; Illeperuma et al., 2021; Jabar, 2017; Nafarzadeh et al., 2017; Panzarella et al., 2017; Zare et al., 2022). According to Table 6, the association of HPV and OLP varied significantly by geographic population; even in different area of the same country, this relationship is associated with varied results (present study; Farhadi et al., 2020; Nafarzadeh et al., 2017; Zare et al., 2022). The type of HPV detected in OLP lesions also seems to be geographically dependent, although high‐risk HPVs, especially HPV‐18, was the predominant subtype in most of the studies (present study; Kaewmaneenuan et al., 2021; T. Liu et al., 2018; Sameera et al., 2019; Vijayan et al., 2022). Although the correlation between HPV and OLP subtype is debatable, most of the studies with significant results report that HPV detection in erosive‐atrophic subtype is well above that of non‐erosive‐atrophic subtype of OLP (Farhadi et al., 2020; Kaewmaneenuan et al., 2021; Vijayan et al., 2022).

**Table 6:** A summary of the most recent studies on the association of human papillomavirus infection and oral lichen planus

Group	First author, year	Country	Specimen	Type of HPV^a^	Method	Number of case/control	Result
Relatively high detection rate (DR) in OLP or significant difference between OLP and control	Vijayan et al. (2022)	India	Biopsy	HPV‐DNA; HPV 16/18	PCR^b^	20/0	DR^c^: 40%; HPV18 was the predominant subtype; the erosive subtype had the greatest DR
	Kaewmaneenuan et al. (2021)	Thailand	Biopsy	16/18	PCR	59/0	DR: 18.6%; HPV18 was the predominant subtype; only detected in atrophic/ulcerative subtype
	Della Vella et al. (2021)	Italy	Biopsy; cytobrush	HPV‐DNA; 6, 11, 16, 53	PCR	52/0	DR: 17%; DR in biopsy: 15%; DR in cytobrush: 6%; HPV6 and 11 was the predominant subtype; HPV 16 detected in only one sample
	Farhadi et al. (2020)	Iran	Biopsy	16/18/33	PCR	32/20	DR: 25% in OLP^d^ and 0% in control; significant association between HPV infection and OLP; detected only in erosive form; HPV‐33 was the predominant subtype (7 out of 8)
	Liu et al. (2018)	China	Biopsy	16/18	IHC^e^	40/24	Significant increase in HPV16/18(E6) protein expression in OLP compared to normal mucosa
	Poulopoulos et al. (2017)	Greece	Cytobrush	HPV‐DNA	PCR	70/48	DR: 12.85% for samples preserved in lysis buffer; DR: 34.28% for samples preserved in DNA•SAL rinse solution; DR: 38.57% in case of dry storage; In OLP lesions, HPV detected significantly more frequently than in control
	Sameera et al. (2019)	India	Biopsy	HPV‐DNA, HPV18	PCR	15/15	DR: 86.6% in OLP and 0% in controls; only HPV 18 detected; no significant association with the type of OLP.
Relatively low detection rate (DR) in OLP or nonsignificant difference between OLP and control	Zare et al. (2022)	Iran	Biopsy	HPV‐DNA	PCR	40/0	DR: 0%
	Bar et al. (2021)	Poland	Biopsy	16/18	IHC	56/0	DR:7.1%; no significant association with the type of OLP
	Illeperuma et al. (2021)	Sri Lanka	Biopsy	HPV‐DNA	PCR	26/30	DR: 0% in OLP and control
	Gomez‐Armayones et al. (2019)	Spain	Biopsy	HPV‐DNA	PCR	41/0	DR: 2.4% (HPV‐DNA was detected only in one OLP sample)
	Nafarzadeh et al. (2017)	Iran	Biopsy	HPV‐DNA	PCR	50/30	DR: 14% in OLP and 3.3% in controls. No significant association between HPV infection and OLP
	Panzarella et al. (2017)	Italy	Cytobrush	HPV‐DNA	PCR and Innogenetics	45/0	DR: 0% in keratotic variants of OLP
	Jabar et al. (2017)	IRAQ	Biopsy	HPV‐DNA	In situ hybridization	30/30	DR: 13.3% in OLP and 10% in controls; No significant association between HPV infection and OLP

^a^ HPV: human papilloma virus.

^b^ PCR: polymerase chain reaction.

^c^ DR: detection rate.

^d^ OLP: oral lichen planus.

^e^ IHC: immunohistochemistry.

From reviewing the recent literature, it can be concluded that some factors may affect the relationship between HPV and OLP, including sampling method (biopsy vs. cytobrush; biopsy possibly has more HPV detection rate than cytobrush; Della Vella et al., 2021), sample storage and preservation (e.g., dry storage had better HPV detection rate than lysis buffer preservation; Poulopoulos et al., 2017) and hyperkeratinization (the presence of hyperkeratosis in keratotic variants of OLP may be a possible cause of difficult detection of HPV; Panzarella et al., 2017).

Considering the oncogenic potential of high‐risk HPVs, it is recommended to perform future studies on dysplastic subtypes of OLPs and on OLP lesions that have progressed to oral squamous cell carcinomas (OSCC; Vijayan et al., 2021). Also, since the HPV‐positive cases were not tested for HPV E6/E7 mRNA expression in most studies, it is recommended to test mRNA expression in future studies to ensure the true role of HPV in OLP etiopathogenesis; it is noteworthy that mRNA expression is considered the gold standard for identification of HPV as a risk factor for oral cancer (Gomez‐Armayones et al., 2019).

The small sample size was the major limitation of this study.

## 5 CONCLUSION

High‐risk HPV can be one of the etiological factors in OLP lesions and possibly the cause of malignant transformation. HPV16 has a greater role in OLP pathogenesis, especially the EA‐OLP subtype, than HPV18. The results show that there is no relationship between sex and HPV in OLP patients. The obtained results require a review of longitudinal studies and more samples.

## AUTHOR CONTRIBUTIONS

Maryam Mohammadi, Hamid Abbaszadeh, Nooshin Mohtasham, and Ebrahim Shafaie have made substantial contributions to conception and design of the study. Maryam Mohammadi, Hamid Abbaszadeh, Nooshin Mohtasham, Hamid Salehiniya, and Ebrahim Shafaie have been involved in data collection and analysis. Hamid Abbaszadeh and Ebrahim Shafaie have been involved in the data interpretation and drafting of the manuscript. Maryam Mohammadi, Hamid Abbaszadeh, Nooshin Mohtasham, Hamid Salehiniya and Ebrahim Shafaie have critically revised the manuscript. All authors have given final approval of the version to be published.

## CONFLICTS OF INTEREST

The authors declare no conflicts of interest.

## Data Availability

All data related to this study are within the text.
